# Two Decades in Plain Sight: Pigmented Basal Cell Carcinoma of the Scalp

**DOI:** 10.7759/cureus.103397

**Published:** 2026-02-11

**Authors:** Sakshi Jaiswal, Umang K Agrawal, Anand K Singh

**Affiliations:** 1 General Surgery, Employees' State Insurance Corporation (ESIC) Hospital, Varanasi, IND

**Keywords:** basal cell carcinoma of scalp, general plastic surgery, pigmented bcc, scalp rotational flap, wound suture

## Abstract

Basal cell carcinoma (BCC) is the most frequently encountered cutaneous malignancy and is characteristically slow-growing with minimal metastatic potential. Pigmented basal cell carcinoma is an uncommon histological variant that may clinically mimic malignant melanoma and other pigmented lesions, often resulting in delayed diagnosis. We report a case of a long-standing pigmented bcc of the parietal scalp that remained indolent for more than two decades in an otherwise healthy male patient. The patient was managed at Employees' State Insurance Corporation (ESIC) Medical College & Hospital, Varanasi, India. The lesion was asymptomatic, with no regional or distant spread. Management consisted of a wide local excision followed by immediate reconstruction using a parieto-occipital scalp rotation flap under general anesthesia. Histopathological examination confirmed pigmented basal cell carcinoma with tumor-free margins. This case highlights the indolent nature of pigmented BCC and underscores the importance of considering this entity in the differential diagnosis of long-standing pigmented scalp lesions.

## Introduction

Basal cell carcinoma (BCC) accounts for approximately 70-80% of all non-melanoma skin cancers and arises from the basal layer of the epidermis. It is strongly associated with cumulative ultraviolet radiation exposure, although genetic predisposition, environmental factors, and immunological status also contribute to its pathogenesis [1,2]. Scalp involvement is relatively uncommon, accounting for approximately 2-5% of all BCCs reported in large clinical series [3]. Despite its lower incidence, BCC of the scalp has been associated with a higher risk of local recurrence compared with other head and neck locations, likely due to delayed clinical detection, complex regional anatomy, and difficulty in achieving adequate surgical margins [4].

Despite being classified as malignant, BCC is characterized by slow growth and an exceptionally low rate of metastasis.

Pigmented BCC is a less common histological variant in which increased melanin production by colonizing melanocytes and accumulation within melanophages impart a dark clinical appearance [5]. This pigmentation may lead to diagnostic confusion with malignant melanoma, pigmented nevi, or seborrheic keratosis. Clinically, pigmented BCC often presents as a well-demarcated, slowly enlarging lesion with shades of brown, black, or blue.

Scalp involvement in BCC is relatively uncommon and poses unique diagnostic challenges. Lesions in this region are frequently concealed by hair, asymptomatic, and therefore detected late in the disease course [6]. Some studies suggest that scalp BCC may demonstrate more aggressive local behaviour due to delayed diagnosis and proximity to vital structures [7]. We present a rare case of pigmented BCC of the parietal scalp with an exceptionally long duration of over 20 years, focusing on its clinical presentation, surgical management, reconstructive strategy, and outcome.

## Case presentation

A male patient presented to the Department of General Surgery at Employees' State Insurance Corporation (ESIC) Medical College & Hospital, Varanasi, India, with a pigmented lesion over the parietal region of the scalp that had been present for approximately 20-25 years. The lesion was insidious in onset and had shown very slow progression over time. The patient denied any associated symptoms such as pain, bleeding, ulceration, discharge, or pruritus. There were no cosmetic or functional complaints.

The patient had no known comorbidities, no history of immunosuppression, and no prior history of malignancy. There was no family history of skin cancer. He reported no symptoms suggestive of regional or distant metastasis, including weight loss, bone pain, or neurological deficits.

**Figure 1:**
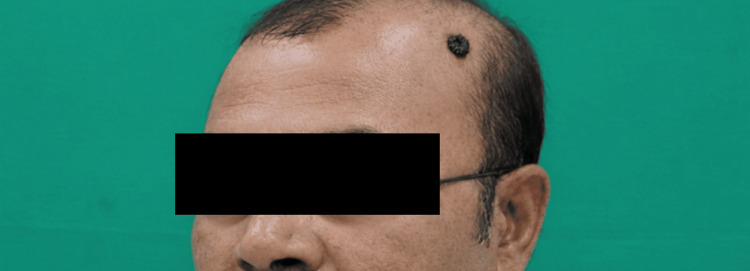
Preoperative clinical photograph showing a long-standing pigmented lesion over the parietal region of the scalp Consent obtained for image use.

On local examination, a well-defined pigmented lesion measuring 8x7 mm was observed over the parietal scalp (Figure 1). The lesion showed dense pigmentation with intact overlying skin and no ulceration. There was no surrounding induration or inflammation. Regional lymph nodes were not palpable, and systemic examination was unremarkable. Differential diagnoses included pigmented BCC, malignant melanoma, pigmented nevus, and seborrheic keratosis. Given the pigmented appearance of the lesion and the initial clinical differential diagnosis, a preoperative incisional biopsy was performed, which was suggestive of pigmented BCC. Based on the histopathological findings and the small size of the lesion, definitive surgical excision with a clinical safety margin of 4 mm was planned prior to excision.

**Figure 2:**
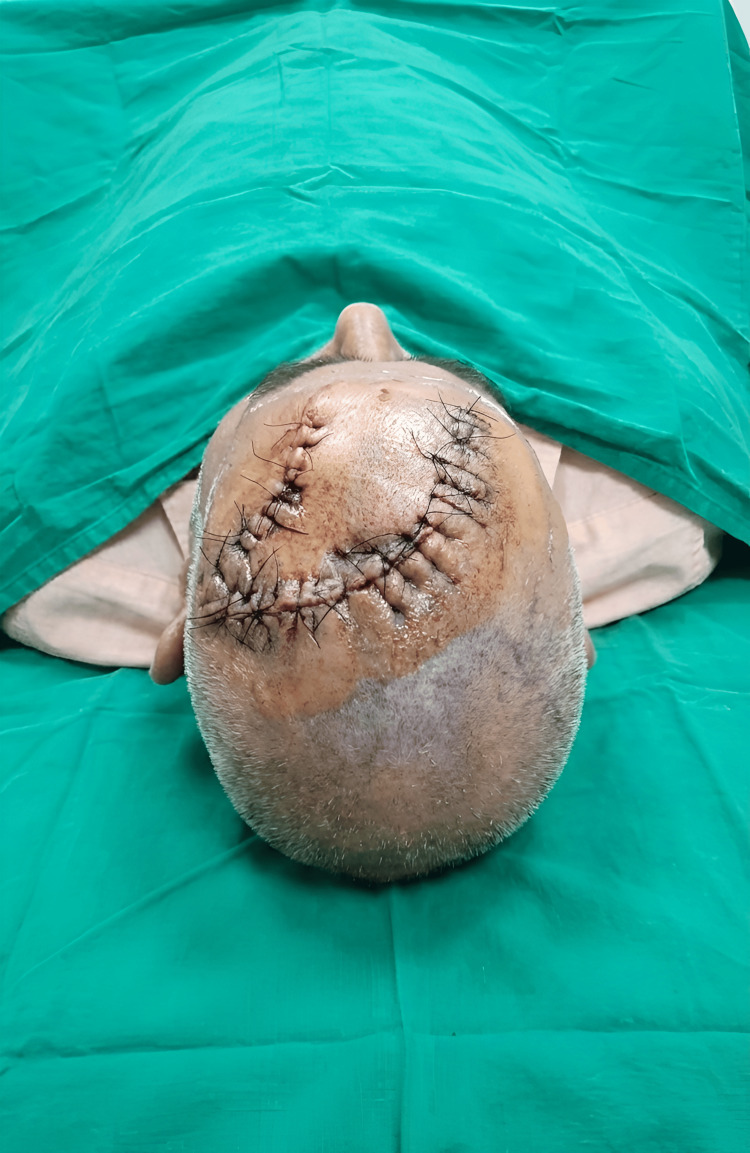
Immediate postoperative photograph following wide local excision of the lesion and reconstruction using a scalp rotation flap Consent obtained for image use.

The patient underwent surgery under general anesthesia. A wide local excision was performed with a peripheral clinical margin of 4 mm. The lesion was excised down to the level of the galea aponeurotica, ensuring adequate deep clearance, and the specimen was submitted (Figure 2). The flap was raised in the subgaleal plane, allowing adequate mobility while preserving vascularity. The flap arc was planned to permit tension-free closure, with an approximate length-to-width ratio of 2:1. Limited galeal scoring was performed to facilitate flap advancement. The flap provided a good tissue match. Hemostasis was secured, and the wound was closed in layers.

**Figure 3:**
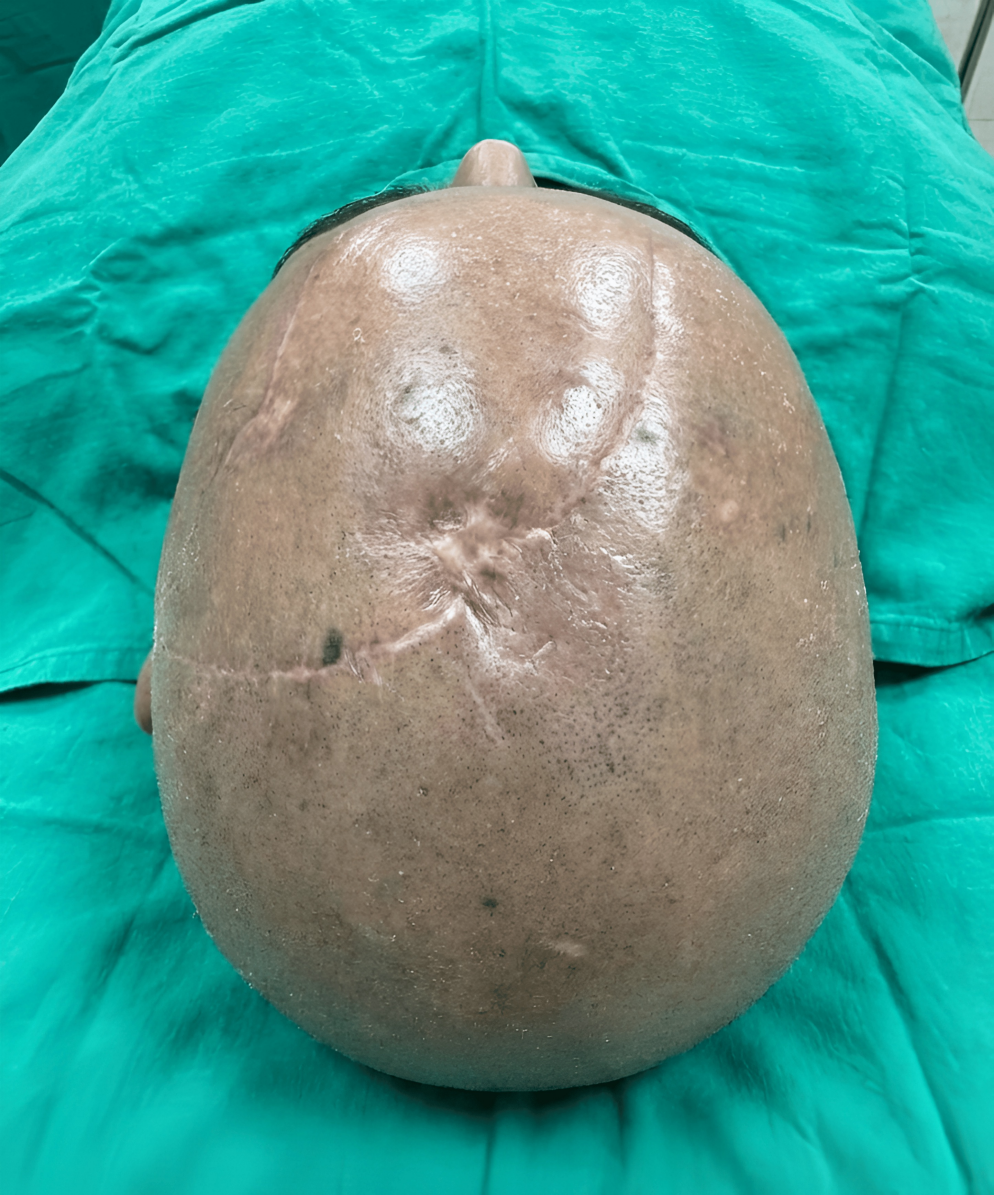
Follow-up clinical photograph at two months demonstrating a well-healed surgical site with good cosmetic outcome and no evidence of recurrence Consent obtained for image use.

The postoperative period was uneventful. The scalp rotation flap remained viable, with no evidence of congestion, necrosis, or wound dehiscence. At two-month follow-up, the surgical site had healed well, demonstrating good contour, acceptable scar maturation, and a satisfactory cosmetic outcome, with no evidence of local recurrence (Figure 3). The patient remains under regular follow-up, currently at a three-monthly interval, and was counseled regarding sun-protective measures.

**Figure 4:**
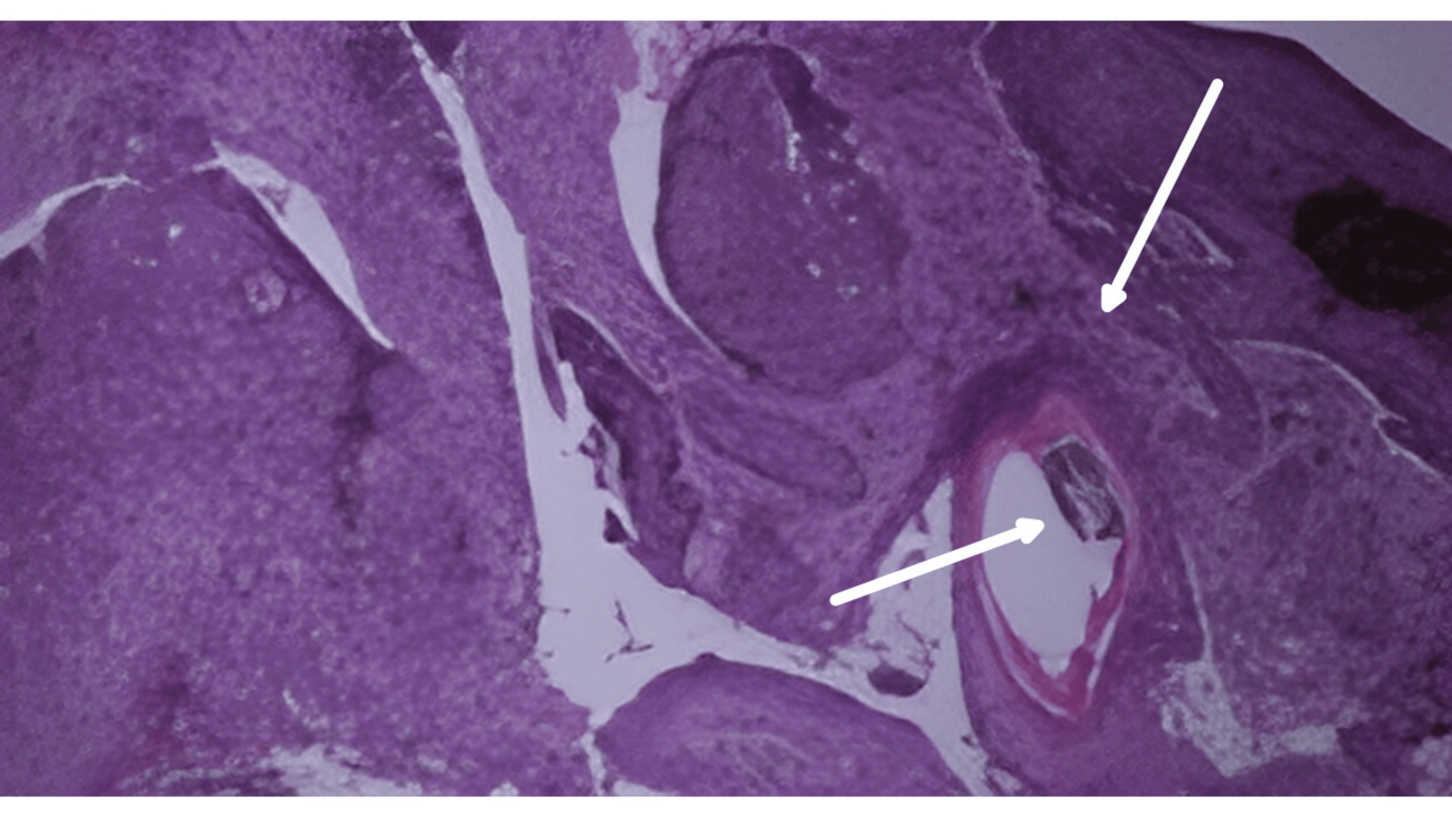
Histopathological image of the excised specimen under hematoxylin and eosin stain revealed nests of basaloid cells with peripheral palisading and abundant melanin pigmentation within the tumor nests and stromal melanophages, confirming the diagnosis of pigmented basal cell carcinoma(×40)

Histopathological analysis of the excised specimen revealed pigmented basal cell carcinoma. All surgical margins were free of tumor (Figure 4).

## Discussion

Pigmented BCC is a recognized histological variant characterized by melanin deposition within basaloid tumor nests and surrounding melanophages, resulting in a clinically dark lesion that may mimic malignant melanoma [5]. In the literature, pigmented BCC accounts for a minority of all BCC cases and is more commonly reported in individuals with darker skin phototypes [5,7]. The present case aligns with previously published reports in demonstrating the indolent biological behavior typical of this subtype.

Most reported cases of pigmented BCC describe lesions evolving over several years; however, the exceptionally prolonged duration of 20-25 years observed in our patient is unusual. Scrivener et al. reported that pigmented BCCs often present earlier due to their striking appearance, whereas non-pigmented lesions may remain unnoticed for longer periods [5]. In contrast, the current case demonstrates that pigmented BCCs located on the scalp can remain clinically silent for decades, likely due to concealment by hair and absence of symptoms. This finding is consistent with observations by Paolino et al., who noted delayed diagnosis of scalp BCCs compared with lesions at more visible anatomical sites [6].

Scalp involvement in pigmented BCC has been variably associated with more aggressive local behavior and higher recurrence rates, particularly when diagnosis is delayed [6,8]. Despite the long-standing nature of the lesion in our patient, there was no clinical or histopathological evidence of aggressive features such as ulceration, perineural invasion, or deep tissue infiltration. However, this finding should be interpreted with caution, as long-standing BCCs of the scalp have been reported to demonstrate aggressive and deeply infiltrative behavior, including involvement of underlying bone and significant local morbidity in delayed presentations. The present case likely represents an indolent, localized variant, underscoring the heterogeneity in biological behavior of pigmented BCC rather than suggesting the benignity of prolonged disease [1,8].

Surgical excision with histologically tumor-free margins remains the gold standard for treatment of BCC [7]. In this case, wide local excision followed by immediate reconstruction using a fronto-parietal rotational scalp flap achieved a satisfactory cosmetic outcome. However, the short follow‑up period limits the ability to comment on long‑term oncologic control. Alternative treatment options described in the literature include Mohs micrographic surgery, which offers superior margin control and lower recurrence rates, particularly for high-risk or recurrent lesions and those located in cosmetically sensitive areas [7]. Mohs surgery could have been considered in the present case; however, given the small lesion size, primary lesion, non‑aggressive histologic subtype, and absence of high-risk features, conventional surgical excision with margin assessment was an appropriate and effective management strategy.

Non-surgical treatment modalities, such as topical imiquimod, photodynamic therapy, or radiotherapy, have been reported for selected cases of superficial or inoperable BCC [2,7]. These options were not pursued in our patient due to the pigmented nature of the lesion, need for definitive histopathological diagnosis, and suitability for complete surgical excision.

In small-to-moderate scalp defects following excision of BCC, several reconstructive options are available, including local advancement flaps, pinwheel flaps, multiple small rotation flaps, or full-thickness skin grafting, particularly when intraoperative margin assessment is not available. While skin grafting remains a reliable option, local flap reconstruction offers advantages in terms of superior color and hair-bearing skin match, maintenance of scalp contour, and reduced risk of secondary alopecia.

In the present case, a fronto-parietal rotation flap was favored due to the relative laxity of the surrounding scalp, robust vascular supply, and the ability to achieve tension-free closure with preservation of hairline and cosmesis. This approach allowed definitive reconstruction in a single stage while maintaining regional anatomy and minimizing donor-site morbidity.

However, reconstruction using a local rotational flap was favored over skin grafting, as supported by Desai et al., who demonstrated superior aesthetic and functional outcomes with local flaps in scalp reconstruction [8].

Overall, this case contributes to the existing literature by highlighting the potential for pigmented BCC of the scalp to remain indolent for decades, emphasizing the importance of clinical vigilance for chronic pigmented scalp lesions, and demonstrating successful management using conventional excision and rotational flap reconstruction.

Limitations

This report describes a single patient, limiting the generalizability of the findings. Long-term follow-up beyond two months was not available at the time of reporting, and, therefore, late recurrence cannot be fully assessed. Moreover, the single, small, non‑aggressive lesion in an otherwise healthy male limits extrapolation to larger, recurrent, or histologically aggressive scalp BCCs.

## Conclusions

Long-standing pigmented BCC of the scalp, although rare, should be included in the differential diagnosis of chronic pigmented scalp lesions. This case illustrates the remarkably indolent nature of pigmented BCC and demonstrates that complete surgical excision with immediate reconstruction can provide an appropriate initial oncologic management and favorable early cosmetic outcomes, with the caveat that longer follow‑up is required to confirm durable control.
